# Effect of adding hip exercises to general rehabilitation treatment of knee osteoarthritis on patients’ physical functions: a randomized clinical trial

**DOI:** 10.1186/s13102-023-00772-7

**Published:** 2023-11-23

**Authors:** Jie Qiu, Tiantian Zhou, Huihong Jin, Yujian Pan, Tingting Qian, Chuan Xue, Wen Xia, Haitao Shi, Bingchen An

**Affiliations:** 1 Department of Rehabilitation, Huadong Hospital Affiliated to Fudan University, Shanghai, China; 2 Qibao Community Health Service Center, Minhang District, Shanghai, China

**Keywords:** Hip exercises, Knee, Osteoarthritis, Rehabilitation, Isometric muscle strengthening, The Western Ontario and McMaster Universities, Numerical Rating Scale, Five times Sit-to-Stand Test, Timed Up and Go Test

## Abstract

**Background:**

Hip adductor and abductor strength were both reduced in KOA patients. But to date, most of the researches have only focused on quadriceps combined with hip abductor strengthening versus quadriceps strengthening.

**Objective:**

The aim of the study is to evaluate the effect of adding hip abductor and adductor strengthening to quadriceps strengthening on lower limb strength, knee pain and physical function in patients with medial compartmental knee osteoarthritis.

**Methods:**

In this study, 42 participants, were randomly divided into two groups: the general treatment group (GT group) and the added-hip-exercise group (AH group). All participants were given a general rehabilitation treatment. The AH group performed hip abductor and adductor strengthening in addition to the general rehabilitation treatment. Knee and hip muscle strength, Five Times Sit-to-Stand Test (FTSST), the Timed Up and Go Test (TUGT), Numerical Rating Scale (NRS), and Western Ontario and McMaster Universities Osteoarthritis Index (WOMAC) scores were assessed at baseline and 6 weeks. A two-sided 2-sample unpaired t test was performed to compare the difference in mean change scores between AH and GT groups.

**Results:**

Finally, 36 participants completed the study: both groups consist of 18 participants. In the per-protocol analysis, the AH group had a greater improvement in knee extension strength (mean changes, 7.84 versus 36.48; *P* < 0.001) and hip abduction strength (mean changes, 5.05 versus 26.62; *P* = 0.001) than the control group. Similarly, the AH group had a greater improvement in the FTSST time (mean changes, 0.40 s versus 3.57 s; *P* < 0.001) and the TUFT time (mean changes, 0.18 s versus 1.67 s; *P* = 0.002) than the GH group. No statistical difference was found in the change of WOMAC pain scores and NRS between the 2 groups.

**Conclusions:**

Older adults with knee OA in the AH group had superior muscle strength, symptoms and daily activity performance at the 6th week than those in the GT group. And adding hip exercises could expedite improvement of pain at the 2th week, but not at the 6th week.

**Trial registration:**

Clinical trial registration numbers and date of registration: ChiCTR-IOR-16009124, Registered 30 August 2016.

## Introduction

Osteoarthritis affects 7% of the global population, which accounts for more than 500 million people worldwide, and has been responsible for 2.2% of total global years lived with disability [1]. More importantly, this increasing trend has been expected to continue in the future due to the process of population aging because of the high cost of the knee replacement procedure, which is a final treatment for end-stage knee osteoarthritis (KOA) [2, 3]. Therefore, it is urgent to find treatments that can slow down osteoarthritis progression and improve symptoms.

According to the Osteoarthritis Research Society International and The European Society for Clinical and Economic Aspects of Osteoporosis and Osteoarthritis guidelines, which were updated in 2019, physical exercise is one of the core treatments for KOA [4]. Many studies have demonstrated a certain decrease in quadriceps strength in participants with KOA [5]. However, the less strength of the quadriceps is, the greater the risk of KOA will be, particularly in women. Thus, quadriceps strengthening can prevent KOA [6]. Further, many studies confirmed that strengthening the quadriceps through exercises can improve life quality and WOMAC physical function and reduce stiffness and pain in participants with KOA, and also delay further development of the disease [7, 8].

In addition to the quadriceps muscle weakness, related studies have shown that patients with KOA also show higher hip muscle weakness than people without KOA [9, 10]. Therefore, hip exercises have recently gained great attention [11, 12]. Several randomized controlled trials studied the effects of hip muscle strengthening exercises on KOA participants’ states. The results showed that compared with participants who did not exercise, participants who performed hip abductor strengthening exercises regularly achieved significant improvements in physical functions and pain reduction [13, 14]. Recent studies suggested that hip adductors might have a higher contribution to functional tasks like balance and walking than quadriceps exercise [15, 16].

To date, most of the researches have only focused on quadriceps combined with hip abductor strengthening versus quadriceps strengthening [11]. But the hip adductor strength was also reduced in KOA patients. According to Andrew et al., both hip abductor and adductor strength have a positive relationship with better physical function [17]. So this research added both hip adductor and hip abductor strengthening to quadriceps exercises.

The lower limb serves as a cooperative whole chain motion, and changes in the hip also affect the knee joint [18]. Thereby, the aim of our study is to evaluate whether the addition of hip adductor and abductor strengthening combined with quadriceps exercises could result in better muscle strength, symptoms and daily activity performance in KOA patients.

## Materials and methods

### Study design

The study conducted a prospective randomized control trial. All baseline and final assessments were performed at the Rehabilitation Department of Huadong Hospital, affiliated with Fudan University Shanghai, China.

We calculated the sample size based on the muscle strength findings in the lower limbs of OA participants, using a medium effect size of 0.31, ɑ of 0.05, and power of 0.95 [19, 20]. The minimum sample size required was 36 participants. Furthermore, regarding the 10% dropout rate, at least 40 participants were enrolled for this study.

### Participant selection

In this study, patients with medial compartmental KOA fulfilling American College of Rheumatology classification criteria were included. Other inclusion criteria were as follows: older than 60 years and have signed informed consent for the study. Further, the exclusion criteria were as follows: 1) received other hip or knee training over the past four weeks; 2) had a history of oral hormone therapy in the past four weeks or a history of intra-articular hormone injections in the past three months; 3) had a hip or knee replacement surgery or another type of hip or knee surgery; 4) Body Mass Index > 36; and 5) accompanied by comorbidities that may affect physical activity, such as neurological and bone, joint, and muscle diseases.

### Patient recruitment

Sixty-seven participants were selected for this study, of which 25 participants were unable to meet the inclusion or exclusion criteria. Finally, 42 participants were enrolled in the study, as shown in Fig. 2. Computer randomly generated a number ranging from one to 42 for each patient; there was no overlapping between the numbers given to the patients. Then, the patients were randomly divided into two groups by simple randomization according to the ratio of 1:1. A staff who was not involved in other parts of the trial completed the randomization before the intervention in the Huadong Hospital. These numbers were put inside sealed and opaque envelopes. Research staff in the hospital then opened the envelope. Per-protocol analysis was performed in the study.

### Intervention

All treatment and exercises were completed in the outpatient clinic at the Huadong Hospital under the supervision of physiotherapists to ensure standardization. A total of two physiotherapists with a mean of six years of clinical experience performed the intervention. The physiotherapists all underwent a one-week training and standardization process.

All participants were engaged in the general rehabilitation treatment for KOA participants, including health education, shortwave, low-level laser therapy, and quadriceps strengthening. The AH group performed hip exercises in addition to the general rehabilitation treatment.

#### Health education

All participants were encouraged to lose weight by dietary intervention; they were informed in detail about the suggested diet plan, the natural course for patients with KOA and about the way how to protect the knee joint.

#### Shortwave

Electrodes were placed symmetrically on the anterior–posterior knee joint; the treatment was performed at the power of 15 W for 20 min once per day, three times per week for six weeks.

#### Low-level laser therapy

Low-level laser therapy was used to irradiate the most painful area, usually in the anterior region of the knee, for eight minutes once per day, three days per week for six weeks.

#### Quadriceps strengthening

**Fig. 1:**
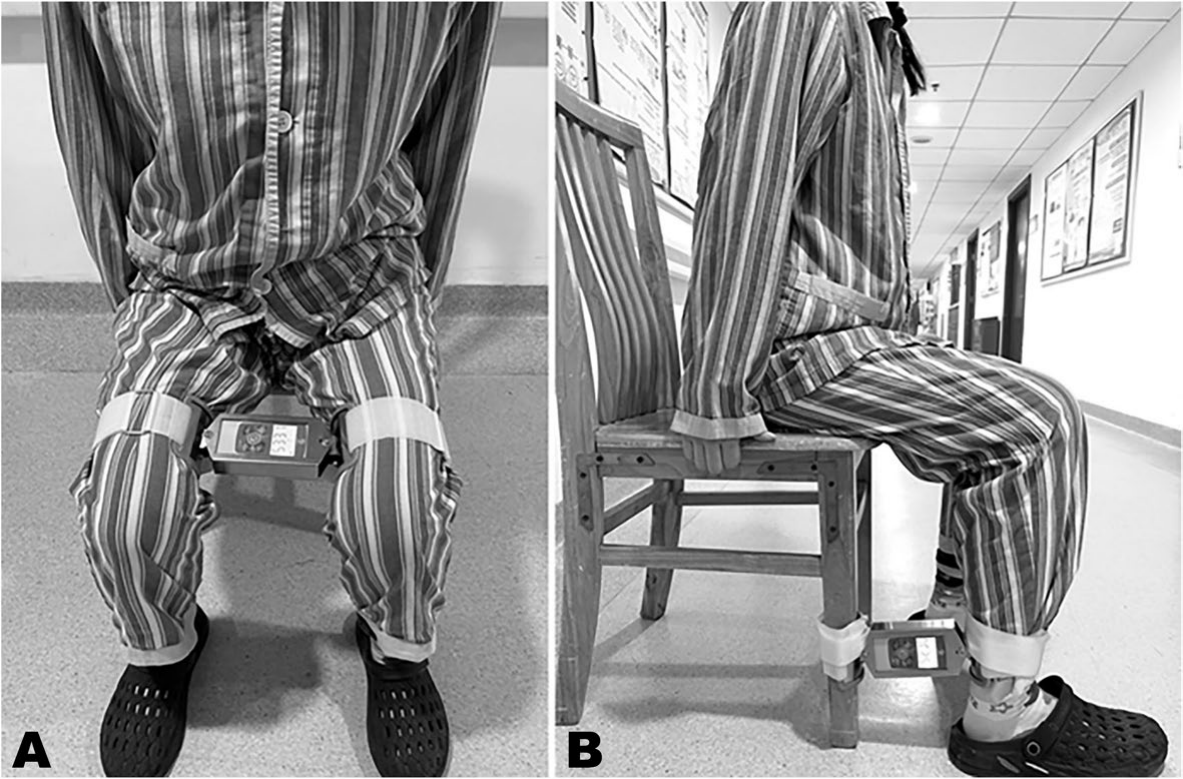
Testing and training position for hip exercises and quadriceps strengthening. **A** Hip muscle training and measurement were completed in the sitting position with the self-developed hip muscle testing and training device tied at 2 cm proximal to the knee joint. Participants performed isometric hip strength training by accomplishing hip abduction and adduction movements. The maximum hip adduction MVIC was displayed on the device screen when participants were squeezing the device by their legs on the inside. Similarly, the maximum hip abduction MVIC was displayed on the device screen when participants were pulling the device by their legs on the outside. **B** Participants completed quadriceps strengthening by taking the sitting position. During exercises, the testing device was fixed above the ankle joint. The maximum knee extensor strength was displayed on the device screen when participants extended their legs forward. The maximum knee flexor strength was displayed on the device screen when participants squeezed the machine backward

The study included the isometric quadriceps contraction training, as shown in Fig. 1B. The knee joint to be tested used 60–80% maximal voluntary isometric contraction (MVIC) to extend the knee, maintaining it for 10-s. Each repetition was separated by a 10-s rest. The training was repeated 10 times in one group, three groups per day, five times per week for six weeks.

#### The hip exercises

Participants took a sitting position and flexed the hip and knee joints at an angle of 90°. Hip muscle exercise was completed before quadriceps strengthening. The two ends of the self-developed hip muscle testing and training device evolved from KAPU dynamometers (KAPU, Germany) were fixed on the opposite lower thighs on both sides, as shown in Fig. 1A. Participants first squeezed the instrument on the inside and then pulled the instrument outward to perform hip adductor and abductor exercises. Each action was performed for 10-s, using 60 – 80% of maximal voluntary isometric contraction. There was a 10-s rest between actions. The training was repeated six to ten times in one group, three groups per day, five times per week for six weeks.

### Outcome scores

The main outcomes were knee extension and flexion strength, hip abductor and adductor strength; the secondary outcomes included the Five Times Sit-to-Stand Test (FTSST), the Timed Up and Go Test (TUGT), Numerical Rating Scale (NRS), and Western Ontario and McMaster Universities Osteoarthritis Index (WOMAC) scores. All data were tested before and after the six-week treatment. The data were collected by another experienced assessor with more than three years of clinical experience blinded to group allocation. The physiotherapist underwent a one-week training and standardization process.

#### Muscle strength test

As mentioned above, the primary outcome of this study included knee extension and knee flexion, hip abductor and hip adductor isometric strength. The MVIC was recorded in Newtons (N). At the knee flexed to 90°, a self-developed hip muscle testing and training device was tied 2 cm proximal to the knee joint, as shown in Fig. 1A. The maximum hip adductor MVIC was displayed on the device screen when patients were squeezing the device by their legs on the inside. The maximum hip abductor MVIC was displayed on the device screen when participants were pulling the device by their legs on the outside. Knee extension and knee flexion force were tested in the same position, but the device was tied above the participants’ ankle joint, as shown in Fig. 1B. Each participant performed the maximal isometric contraction to obtain knee extension and knee flexion force. The maximal effort was ensured through verbal encouragement.

#### FTSST

In the elderly, the FTSST result has a strong relationship with lower-limb strength and functionality since it includes a common activity people perform daily [21, 22]. Participants sat on an armless chair, with a sitting part at the height of 43 cm. Participants were required to place their feet on the floor and cross their hands on their chests. Their backs should not recline or attach to the back of the chair. After hearing the command to start the test, participants should stand up and then sit down five times at the fastest speed they could achieve. Then, the time it took for participants to perform the entire course was recorded. During the test, participants were continuously given verbal encouragement when necessary but were not helped in performing the exact test action [23]. The test was repeated three times with breaks of 30 s; the results were averaged and saved as a final result.

#### TUGT

According to Shimada et al., [24] the TUGT is strongly associated with the walking speed of elderly women with KOA. When the time to complete the TUGT is shorter, participants have higher functional mobility and a lower risk of falling [25]. In this test, participants sat on an armchair with a sitting part at the height of 46 cm. In addition, a marker or brightly colored ribbon was used to make a mark on the ground 3 m away from the chair to ensure that participants could see it. After the command “start” was given, participants immediately stood up from the chair, walked forward 3 m (till they reached the marked place) at the fastest speed they could achieve, and then turned to sit back on the chair. The time between the command to start was given until participants returned and sat on the chair was recorded [26]. This test was performed twice, and the average result was saved as a final result.

#### NRS

The NRS has been commonly used to assess pain intensity. In this test, a straight line was equally divided into 10 parts, and a total of 11 numbers from zero to 10 were used to indicate different pain levels in patients [27]; zero indicated no pain, and 10 indicated pain that was too severe to endure. Participants were asked to provide a mark on the horizontal line according to their subjective feelings to describe the pain level. The NRS had high responsiveness and was convenient for recording [28].

#### WOMAC score

The WOMAC is a scoring scale specially developed for hip and knee osteoarthritis. Its function description is mainly focused on the lower limbs [29]. The WOMAC score is typically given based on participants’ clinical symptoms and the corresponding signs to assess the severity of the disease and the efficiency of treatment. In this study, the WOMAC scale was used to evaluate the functional status of lower extremities suffering from KOA by assessing 17 functional ADLs, five pain-related activities, and two stiffness categories [30]. The WOMAC index referred to 24 parameters, including pain (score range 0–20), stiffness (score range 0–8), and functionality (score range 0–68); it should be noted that higher scores indicated worse symptoms. The research of Symond [31] has shown that the WOMAC scale is widely used in the function of Chinese KOA participants due to its objective reliability, validity, and sensitivity.

Adherence to treatment was assessed by the total number of treatment sessions performed in 6 weeks. Adverse effects and adherence were recorded by physiotherapists.

### Statistical analyses

Statistical analyses were conducted using SPSS 20 (IBM SPSS Inc., Chicago, USA). In the first step, we tested to see if continuous variables were normal (Shapiro–Wilk normality test), and they were expressed in the form of $\overline{x }$(s), indicating mean (standard deviation). The Shapiro–Wilk normality test was performed when the variance was homogeneous; otherwise, the difference between post- and pre-treatments was determined by an independent sample t-test to reduce the calculation error. We adopted the per-protocol analysis. A two-sided 2-sample unpaired t test was performed to compare the difference in mean change scores of hip abductor and adductor strength, knee extension and flexion strength, FTSST, TUGT, NRS, and WOMAC scores between AH and GT groups. Data on counts like female were expressed as *n* (%) and compared by the Chi-square test, and a difference of *P* < 0.05 was considered statistically significant.

### Funding source role

The funders played no role in the design, conduct, or reporting of this study.

## Results

**Fig. 2:**
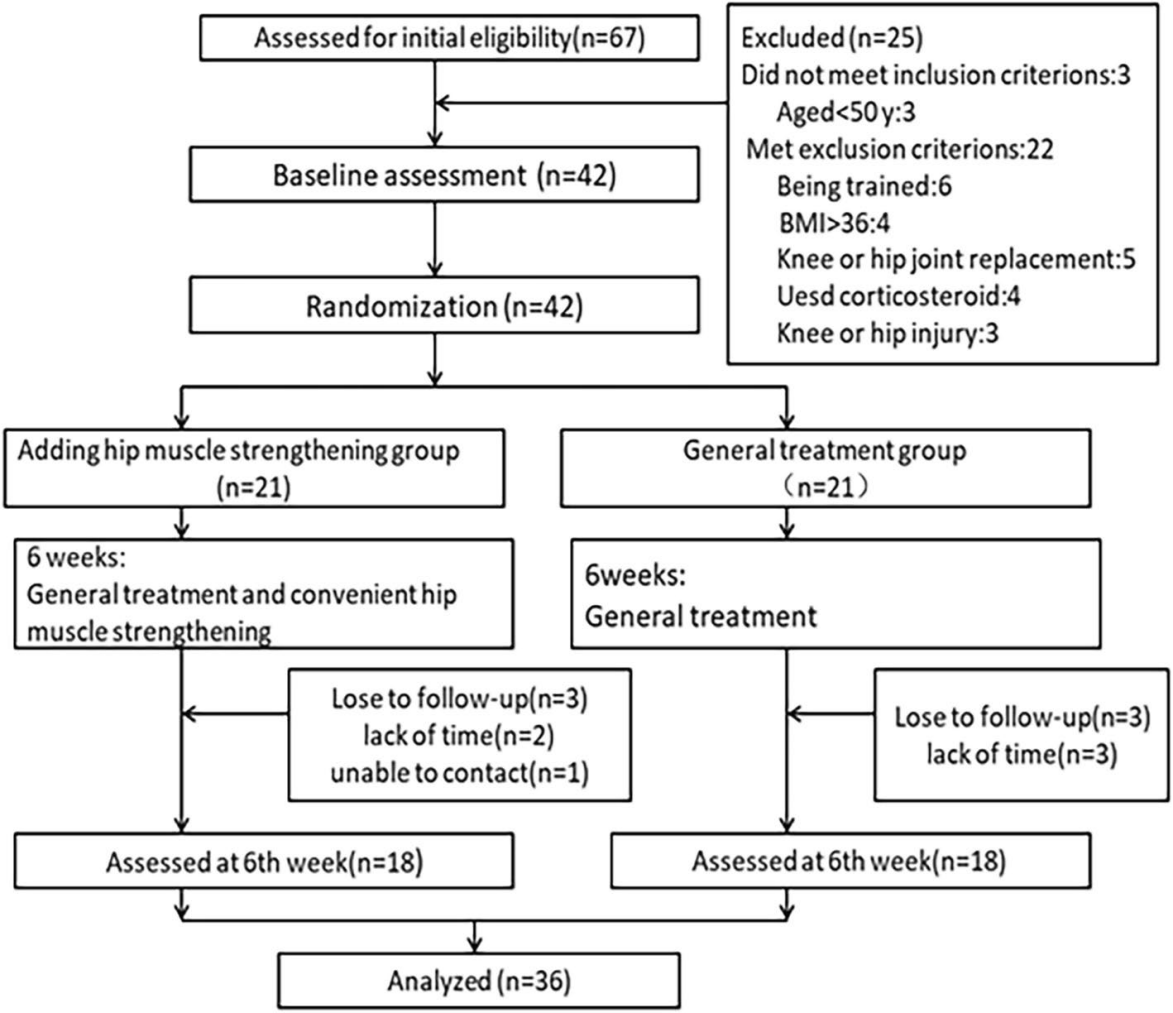
Flow diagram

The average age of the subjects was 72.84 (6.34) years. Of a total of 42 participants, 36 participants completed the study, as shown in Fig. 2, and no adverse events occurred during the study period. Although six participants withdrew from the trial due to personal reasons, none of them had the side effect of pain. Namely, three participants from the GT group and two participants from the AH group withdrew from the study because they did not have enough time to finish it, and one patient from the AH group did not attend the follow-up appointments. Apart from these participants, all the other participants completed all the sessions. No minor adverse event was reported in both groups. The basic conditions of the two groups’ participants are presented in Table 1. The baseline demographic data of the two groups did not differ in terms of age, gender, weight, height, BMI, and course of the disease, as shown in Table 1.

**Table 1:** Demographic data

	GT group (*n* = 18)	AH group (*n* = 18)
Age (yr)	74.39 (4.81)	71.28 (7.84)
Female (n%)	14.0 (77.8)	13.0 (72.2)
Height	160.06(9.12)	162.21 (7.84)
Weight	62.61 (6.59)	64.78 (12.96)
BMI (kg/m^2^)	24.74 (3.11)	24.47 (3.41)
Course of disease (yr)	4.50 (4.32)	4.17( 4.84)
Bilateral/unilateral, n/n	8/10	9/9
K-L grade, (n)		
II	10	9
III	7	8
IV	1	1
Previous drug treatment, (n)		
Acetaminophen	3	3
Nonsteroidal anti- inflammatory drugs	2	3
Glucosamine or chondroitin products	6	8

Counting data was expressed by composition ratio (n%) or total number (n)

Measurement data was all expressed by mean (standard deviation)

*GT Group* General treatment group, *AH Group* Hip muscle exercises added to the general treatment, *BMI* Body mass index

As shown in Table 2, the AH group significantly increased the knee extension strength (36.48 (25.43) N versus 7.84 (6.54) N, having *P* < 0.001), knee flexion strength (15.19 (12.45) N versus 8.60 (10.13) N, having *P* = 0.033), hip abduction strength (26.62 (18.67) N versus (5.05 (17.98) N, having *P* < 0.001), and hip adduction strength (27.49 (23.71) N versus 3.43 (20.47) N, having *P* = 0.001) compared with the GT group.

**Table 2:** Comparison results of knee extension and flexion strength, hip adduction and abduction strength changes between the GT and AH groups

	GT group (*n* = 18)			AH group (*n* = 18)			Difference between groups
	Week 0	Week 6	Week 6-Week 0	Week 0	Week 6	Week 6-Week 0	*P* value
Knee extension strength (N)	84.93 (31.58)	93.54 (29.59)	7.84 (6.54)	81.03 (17.50)	117.49 (34.46)	36.48 (25.43)	< 0.001
Knee flexion strength (N)	60.87 (16.55)	68.71 (16.08)	8.60 (10.13)	65.77 (13.98)	80.96 (21.76)	15.19 (12.45)	0.033
Hip abduction strength (N)	113.95 (32.48)	119.02 (41.64)	5.05 (17.98)	131.05 (73.65)	157.67 (74.46)	26.62 (18.67)	0.001
Hip adduction strength (N)	125.38 (33.73)	128.82 (37.95)	3.43 (20.47)	125.44 (51.64)	152.93 (57.82)	27.49 (23.71)	< 0.001

Mean (standard deviation) is given for each parameter

*GT Group* General treatment group, *AH Group* Hip muscle exercises added to the general treatment

Compared with the GT group, participants in the AH group had statistically and significant improvements in the TUGT value (− 1.67 (1.28) s versus − 0.18 (1.45) s; having *P* = 0.002) and FTSST value (− 3.57 (2.78) s versus − 0.40 (1.46) s, *P* < 0.001), as shown in Table 3.

**Table 3:** Comparison results of the FTSST, TUGT, NRS, and WOMAC changes between the GT and AH groups

	GT group (*n* = 18)			AH group (*n* = 18)			Difference between groups
	Week 0	Week 6	Week 6-Week 0	Week 0	Week 6	Week 6-Week 0	*P* value
FTSST time (s)	24.16 (17.21)	23.77 (16.82)	0.40 (1.46)	24.67 (15.89)	21.06 (15.10)	3.57 (2.78)	< 0.001
TUGT time (s)	13.99 (5.96)	13.81 (5.85)	0.18 (1.45)	14.19 (6.99)	12.51 (6.35)	1.67 (1.28)	0.002
NRS	4.78 (1.90)	3.50 (1.86)	1.28 (1.02)	3.50 (1.86)	3.17 (1.58)	1.50 (1.47)	0.601
WOMAC pain	5.50 (3.59)	4.50 (3.09)	1.00 (0.77)	5.11 (3.86)	3.67 (3.24)	1.44 (1.42)	0.252
WOMAC stiffness	2.06 (1.89)	1.78 (1.86)	0.28 (0.67)	2.50 (1.86)	1.33(1.41)	1.17 (0.86)	0.001
WOMAC function	17.22 (9.92)	16.00 (10.07)	1.22 (1.06)	19.72 (12.36)	15.44 (11.65)	4.28 (2.63)	< 0.001

Mean (standard deviation) is given for each parameter

*GT Group* General treatment group, *AH Group* Hip muscle exercises added to the general treatment, *FTSST* Five Times Sit-to-Stand Test, *TUGT* Timed Up and Go Test, *NRS* Numerical Rating Scale, *WOMAC* Western Ontario and McMaster Universities (WOMAC) Osteoarthritis Index

Both the NRS and the WOMAC pain scores of the AH group decreased more than those of the participants in the GT group after six weeks of treatment (− 1.50 (1.47) versus − 1.28 (1.02), *P* = 0.601), but there was no significant difference between the groups, as shown in Table 3.

All subscales of the WOMAC scores improved more in the AH group than in the GT group (stiffness subscale: − 1.17 (0.86) versus − 0.28 (0.67), *P* = 0.001; function subscale: − 4.28 (2.63) versus − 1.22 (1.06), *P* < 0.001). Most of these subscales, except for the pain scores, decreased significantly, as shown in Table 3.

## Discussion

The results showed that additional hip exercises added to the general treatments could increase muscle strength and lower-limb function of the elderly with KOA.

In the AH group, hip abductor strength and quadriceps strength were increased by 20.32% and 45.02%. Jianxiong Wang et al. found that after conducting six-week-quadriceps-combined-hip-abductor strengthening, hip abductor strength and quadriceps strength differed by 15.55% and 14.9% [32]. After adding hip adduction exercise, more improvement in hip abductor strength and quadriceps strength was found in KOA patients. A potential reason for better outcomes could be explained by arthrogenic muscle inhibition (AMI). Namely, as a consequence of KOA, the quadriceps muscles are unable to contract fully, which has been known as AMI [33, 34]. Previous research has shown that hip exercises can effectively activate the quadriceps muscle without causing additional knee pain [35]. Therefore, by performing hip exercises after quadriceps strengthening, the quadriceps can be further activated. Thus, through reducing quadriceps weakness, additional hip exercises can help to mitigate the AMI effect.

AH group resulted in greater improvements in FTSST time, TUGT time and WOMAC function score than GT group. This may because as hip and knee muscle strength increased, the lower-limb function could be achieved by the participants. According to Chun et al., muscle strength was the only modifiable factor that was correlated with physical performance, such as FTSST and TUGT, irrespective of the radiographic severity of KOA [36]. Specifically, patients in the AH group reported a clinically and statistically meaningful decrease in FTSST time at the 6-week treatment [37]. The reason for AH group gained more improvement in FTSST time may attribute to the additional hip abduction and adduction exercises. The strength of proximal hip muscles are associated with pelvis and trunk stabilization. Namely, they are related to the improvements in the balance function of KOA participants and can reduce the FTSST time [38, 39]. The sit-to-stand action is an essential movement in people’s daily lives, which requires achieving balance by controlling the center of gravity of the human body. Therefore, with the improvement in the FTSST value, participants will have fewer abnormalities in balance and a lower risk of falling [40].

Although the AH group showed greater improvements in pain reduction than the GT group, no clinically and statistically important improvement was found in the AH group compared to the GT group. It should be noted that previous research simply compared hip muscle strengthening with quadriceps strengthening, and the early effect of hip muscle strengthening in reducing knee pain was significantly better than that of quadriceps strengthening in the two-week period [35]. This result was consistent with that of Yuenyongvivat et al. [41], who concluded that adding hip exercises provided a faster improvement in pain relief. However, after 2–4 weeks, no differences were found in pain relief between the groups. This could indicate that adding hip exercises had a limited effect on pain relief in the long term. According to the results of shortwave [42, 43], low-level laser therapy [44] and education [45] have a positive effect on pain relief. Thus, it has been hypothesized that general rehabilitation treatment combined with quadriceps strengthening could have a ceiling effect in pain relief in two weeks. Another potential reason for not finding clinical significance in pain relief could be the small number of cases.

Although the AT group performed additional hip exercises, the adherence rate of the two groups showed no difference. This could be because hip exercises were easy to understand. In contrast, exercises that were too complex for participants to understand might result in adherence obstacles [46]. Finally, hip exercises were conducted in the sitting position to reduce the risk of falling to make the exercises convenient to perform for participants of all ages, especially the elderly.

A limitation of this study is the short observation period. In the future, it would be necessary to extend the observation period, conduct multi-center randomized controlled studies, increase the number of cases, and further explore related mechanisms. As for future works, it would be good to examine the effect of different difficulty levels of hip exercises and their periodical repetition. Namely, after a certain period, the body stops reacting to the same type and level of exercises, so maybe increasing the repetition times or changing the types of exercises could provide a longer effect. Also, it would be interesting to analyze the effect of these types of exercises on younger patients with diagnosed KOA for a longer period.

## Conclusions

Both hip abductor and adductor exercises should be strongly recommended as a part of the exercise prescription for KOA patients.

## Data Availability

All data are available from the corresponding author, An Bingchen.
